# *Snca* and *Bdnf* Gene Expression in the VTA and Raphe Nuclei of Midbrain in Chronically Victorious and Defeated Male Mice

**DOI:** 10.1371/journal.pone.0014089

**Published:** 2010-11-23

**Authors:** Natalia N. Kudryavtseva, Natalia P. Bondar, Ul'yana A. Boyarskikh, Maxim L. Filipenko

**Affiliations:** 1 Institute of Cytology and Genetics SD RAS, Novosibirsk, Russian Federation; 2 Institute of Chemical Biology and Basic Medicine SD RAS, Novosibirsk, Russian Federation; National Institutes of Health, United States of America

## Abstract

**Background:**

Alpha-synuclein (α-Syn) is a small neuronal protein that has been found to be expressed throughout the brain. It has been shown that α-Syn regulates the homeostasis of monoamine neurotransmitters and is involved in various degenerative and affective disorders. There is indication that α-Syn may regulate expression of the brain-derived neurotropic factor (BDNF) which plays an important role in the mood disorders.

**Methodology/Principal Findings:**

The study aimed to analyze the mRNA levels *of Snca* and *Bdnf genes* in the ventral tegmental area (VTA) and raphe nuclei of the midbrain in male mice that had each won or defeated 20 encounters (20-time winners and 20-time losers, respectively) in daily agonistic interactions. Groups of animals that had the same winning and losing track record followed by a no-fight period for 14 days (no-fighting winners and no-fighting losers) were also studied. *Snca* mRNA levels were increased in the raphe nuclei in the 20-time losers and in the VTA of the 20-time winners. After no-fight period *Snca* mRNA levels decreased in both groups. *Snca* mRNA levels were similar to the control level in the VTA of the 20-time losers and in the raphe nuclei of the 20-time winners. However *Snca* gene expression increased in these areas in the no-fighting winners and no-fighting losers in comparison with respective mRNA levels in animals before no-fight period. *Bdnf* mRNA levels increased in VTA of 20-time winners. Significant positive correlations were found between the mRNA levels of *Snca* and *Bdnf* genes in the raphe nuclei.

**Conclusions/Significance:**

Social experience affects *Snca* gene expression depending on brain areas and functional activity of monoaminergic systems in chronically victorious or defeated mice. These findings may be useful for understanding the mechanisms of forming different alpha-synucleinopathies.

## Introduction

Alpha-synuclein (α-Syn) is a small neuronal protein localized in the presynaptic compartment of neurons that has been found to be expressed throughout the brain [1], [2]. It has been shown that α-Syn regulates the homeostasis of monoamine neurotransmitters, through its trafficking, and regulation of the cell surface expression and, thereby, the activity of dopamine, serotonin and norepinephrine transporters [3]–[5]. α-Syn is involved in various degenerative disorders such as Parkinson's disease, dementia with Lewy bodies [review 1, 3, 6, 7], which are recognized as alpha-synucleinopathies. It is suggested that α-Syn may also play a pathophysiological role in depressive symptoms [8], [9].

Previous data have revealed that long positive or negative fighting experience in daily agonistic interactions in male mice is accompanied by different changes in brain neurochemical activities. It has been experimentally demonstrated that repeated aggression which is accompanied by victories, leads to total activation of brain dopaminergic systems. This activation was detected in the winners as elevated DOPAC (3,4-dihydroxyphenyleacetic acid) levels or/and increased DOPAC/DA (dopamine) ratios in different brain areas [10], [11]. Enhanced expression of the *Th*, *Dat1* and *Snca* genes [12], [13] , which are associated with brain dopaminergic systems was shown in the ventral tegmental area (VTA) of the winners. Reduced activity of brain serotonergic system was suggested to be developed under repeated experience of aggression as has been shown by decreased tryptophan hydroxylase activity in some brain areas [14]–[16]. Daily social defeats are accompanied by activation of serotonergic system as shown by changes of serotonin and 5-hydroxyindoleacetic acid levels in different brain areas of the losers [10], [17] and enhanced expression of *Sert* and *MaoA* genes in the raphe nuclei of the midbrain [18]. Reduced activity of brain dopaminergic systems was supposed in defeated animals [17].

The paper aimed to study the possible changes of *Snca* gene expression in animals with different brain monoaminergic activity. We focused on the VTA containing the cell bodies of mesolimbic dopaminergic neurons, because mesolimbic dopaminergic projections from the VTA play an important role in the mediation of rewarding processes [19] which is supposed to accompany positive fighting experience [20], [21]. The raphe nuclei containing the cell bodies of serotonergic neurons whose projections are involved into regulation of stress, depression, anxiety and in many kinds of social behaviors was also studied.

The brain-derived neurotrophic factor (BDNF) is associated with many psychiatric disorders such as depression and anxiety [22], [23] by participating in differentiation, growth and maintenance of neuronal cells. Differently directed changes BDNF and α-Syn were revealed in the pathogenesis of Parkinson's disease affecting dopaminergic systems [7]; [24]. Moreover there was indication that overexpression of α-syn may decrease BDNF expression [25]. It was supposed that *Bdnf* gene together with *Snca* gene in the VTA and raphe nuclei of midbrain may be involved in the consequences of repeated agonistic interactions. Expression of *Bdnf* gene was also studied in the VTA and raphe nuclei of midbrain in the mice before and after period of repeated agonistic interactions.

## Materials and Methods

### Animals and housing

Adult male mice of the C57BL/6J strain from a stock maintained in the Animal Facility of the Institute of Cytology and Genetics, SD RAS, (Novosibirsk, Russia) were used. The animals were housed under standard conditions (12∶12 h light/dark regime, switch-on at 8.00 a.m.; food (pellets) and water available *ad libitum*). Mice were weaned at one month of age and housed in groups of 8–10 in plastic cages (36×23×12 cm). Experiments were performed on mice 10–12 weeks of age.

### Ethics Statement

All procedures were in compliance with the European Communities Council Directive of November 24, 1986 (86/609/EEC). This study was approved by Scientific Council N 9 of the Institute of Cytology and Genetics SD RAS of March, 24, 2010, N 613.

### Chronic agonistic interactions (winners and losers)

Aggressive or submissive behaviors in male mice were induced using the sensory contact model [26]. Pairs of weight-matched animals were each placed in a steel cage (28×14×10 cm) bisected by a perforated transparent partition allowing the animals to see, hear and smell each other, but preventing physical contact. The animals were left undisturbed for three days to adapt to new housing conditions and sensory contact before they were exposed to encounters. In the second half of the light period, the lid was replaced by a transparent one and five minutes later the partition was removed for 10 minutes to encourage agonistic interactions. The superiority of one of the mice was firmly established within two or three encounters (three days) with the same opponent. The superior mouse would be attacking, biting and chasing another, who would be displaying only defensive behavior (sideways postures, upright postures, withdrawal, lying on the back or freezing). As a rule, in our experiments, aggressive confrontations between males are discontinued by lowering the partition if the aggression has lasted more then 3 min or less. Each defeated mouse (loser) was exposed to the same winner for three days, while afterwards each loser was placed, once a day after the fight, in an unfamiliar cage with an unfamiliar winner behind the partition. Each victorious mouse (winner) remained in its original cage. This procedure was performed for 20 days and yielded an equal number of winners and losers.

Five groups of animals were used. (1) 20-time winners: a group of mice that had each won 20 encounters in succession (2) No-fighting winners: a group of 20-time winners who were allowed to live for 14 days after the last encounter without agonistic interactions; (3) 20--time losers: a group of mice that had each defeated 20 encounters; (4) No-fighting losers: a group of 20-time losers who were allowed to live for 14 days after the last encounter without agonistic interactions. During the no-fight period animals shared a cage with a partner (losers or winners); the partition between their compartments being down at all times, to prevent encounters. (5) Controls: the mice that had been housed individually for five days. The rationale for this choice is that it gives the best trade-off between group housing and social isolation: five days is sufficient for group housing to no longer be a factor and insufficient for social isolation to become a factor. Special investigations confirmed strong rationality of this control in the sensory contact model [27]. Each experimental group contained 7–13 animals.

To measure mRNA levels in the brain areas, all the mice were decapitated simultaneously: 20-time winners and 20-time losers, 24 hours after the last agonistic interaction; no-fighting winners and no-fighting losers, immediately after 14-day no-fight period; and the controls, on day 6 of individual housing. The mouse brains were removed and chilled rapidly on ice. The VTA and raphe nuclei were dissected according to the Mouse Brain Atlas [28]. Obtained tissue was rapidly frozen in liquid nitrogen and stored at −70°C until used.

#### Total RNA extraction and reverse transcription

Total RNA was extracted from each individual brain tissue sample using the Chomczynski and Sacchi method [29] with modifications. Total RNA was quantified by measuring the absorbance at 260 nm. The integrity of total RNA was assessed by agarose gel electrophoresis. 1 µg of total RNA was used for cDNA synthesis by MoMLV reverse transcriptase (Biosan, Novosibirsk, Russia).

### Real-time quantitative PCR

Amplification was performed using an iQ5 iCycler (Bio-Rad, Hercules, CA, USA). *Bdnf*, β-actin (*Actb*), and cyclophilin (*Cphn*) mRNA levels were quantified by TaqMan real-time PCR. PCR was performed in a total volume of 25 µl containing an aliquot of the RT mixture, dNTPs, the appropriate concentrations of sense and anti-sense primers, a TaqMan probe, PCR buffer, and hot-start Taq DNA polymerase (Biosan, Novosibirsk, Russia). Amplification was run for 2 min at 96°C, followed by 37 cycles of 15 s at 96°C, 45 s at 61°C. Fluorescence was monitored for 10 s after the last cycle.

*Snca* mRNA levels were quantified by Sybr Green I real-time PCR in a total volume of 25 µl containing an aliquot of the RT mixture, dNTPs, the appropriate concentrations of the sense and anti-sense primers, Sybr Green I (Invitrogen), PCR buffer, and hot-start Taq DNA polymerase. Amplification was run for 3 min at 95°C, followed by 40 cycles of 10 s at 92°C, 6 s at 60°C, 6 s at 72°C and 10 s at 85°C. Fluorescence was monitored for 10 s after the last cycle. To check for the presence of non-specific PCR products or primer-dimers, a melting curve analysis was performed after the final PCR cycle.

Amplification efficiencies were calculated a relative standard curve derived from fourfold serial dilutions of pooled cDNA. In all cases, the amplification efficiency was higher than 85%. Each sample was PCR-amplified twice. RT-PCR results were quantified using the relative standard curve method. The level of expression of each gene was normalized to the mean level of expression of the *Actb* and *Cphn* genes. The oligonucleotide primers and probes were designed using Beacon Designer 5.0 (PREMIER Biosoft International, USA). The PCR primer and probe sequences are shown in Table 1.

**Table 1:** Primers and probe sequences.

Genes	Primer and probe sequences	
*Bdnf*	Sense	5′-ACTATGGTTATTTCATACTTCGGTT-3′
	anti-sense	5′-CCATTCACGCTCTCCAGA-3′
	Probe	5′-FAM-CGTCCACGGACAAGGCAACTT-BHQ1-3′
*Snca*	Sense	5′-TGACAGCAGTCGCTCAGA-3′
	anti-sense	5′-CATGTCTTCCAGGATTCCTTC-3′
*Cphn*	Sense	5′-GAGAACTTCATCCTAAAGCATACAG-3′
	anti-sense	5′-TCACCTTCCCAAAGACCA-3′
	Probe	5′- TAMRA -CGTTGCCATCCAGCCATTCAG-BHQ2-3′
*Actb*	Sense	5′- TCTTTGCAGCTCCTTCGTT -3′
	anti-sense	5′-CGATGGAGGGGAATACAG-3′
	Probe	5′- ROX-CACACCCGCCACCAGTTCGC-BHQ2-3′

#### Statistical analysis

The data are reported as mean ± SEM. Two-way ANOVA of ranked data was used to reveal effect of factors “social status” (20-time winners and 20-time losers) and “no-fight period” and its interactions. Statistical analysis of data was also performed using the Kruskal-Wallis one-way analysis of variance (ANOVA) with factor “groups” in consideration – the control, 20-time winners, 20-time losers, no-fighting winners and no-fighting losers. A post-hoc pair-wise comparison of the groups was made with the Mann-Whitney *U* test. Correlations were assessed using Spearman's rank correlation coefficient. We searched for correlations between the *Bdnf* and *Snca* mRNA levels in each experimental group separately and in combination. The statistical significance was set at P≤0.05; the trend level was set at 0.05<P<0.1.

## Results

For the *Snca* mRNA levels, two-way ANOVA for ranked data reports a significant interaction for “social status” and “no-fight period” factors in the raphe nuclei (F(1,47) = 15.57, *p*<0.001) and in the VTA (F(1,33) = 15.87, *p*<0.001). For the *Bdnf* mRNA levels no significant interactions were found between social status and no-fight period effects in the raphe nuclei (F(1,47) = 1.02, NS) and in the VTA (F(1,31) = 0.44, NS).

Kruskal-Wallis analysis revealed a significant influence of the factor “groups” for the *Snca* mRNA level in the VTA (H (4, N = 44) = 14.41, *p*<0.01) and in the raphe nuclei (H (4, N = 64) = 13.69, *p*<0.01) as well as for the *Bdnf* mRNA level in the raphe nuclei (H (4, N = 64) = 10.58, *p*<0.05). There was no significant influence of the factor “groups” on the *Bdnf* mRNA level in the VTA (H (4, N = 42) = 2.96, NS).

**Figure 1:**
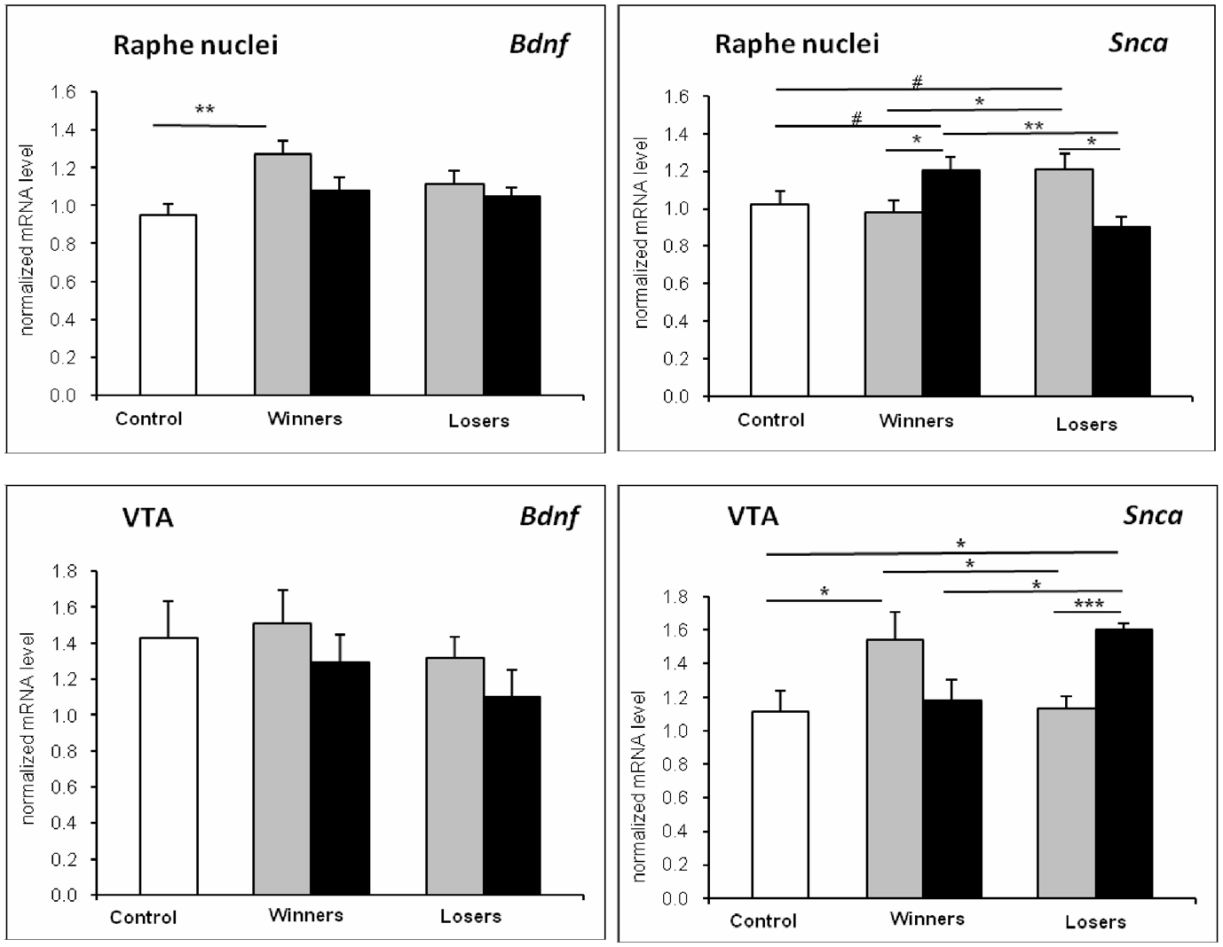
The normalized *Snca* and *Bdnf* mRNA levels in the VTA and raphe nuclei of midbrain of the controls (white columns), 20-time winners and 20-time losers (grey columns) and no-fighting winners and no-fighting losers (black columns). * *p*<0.05; ** *p*<0.01; *** *p*<0.001; # - 0.05<*p*<0.1 comparisons between respective groups.

Based on the Mann-Whitney *U* test (Figure 1), in the VTA, the 20-time winners and no-fighting losers had increased *Snca* mRNA levels as compared to the control (U = 16; *p*<0.05 and U = 7; *p*<0.05, respectively). Groups of the 20-time winners and 20-time losers as well as groups of the no-fighting winners and no-fighting losers differed significantly (U = 31; *p*<0.05 and U = 8; *p*<0.05, respectively). No-fighting losers had increased *Snca* mRNA levels as compared to the 20-time losers before no-fight period (U = 2; *p*<0.001).

In the raphe nuclei, the no-fighting winners had increased *Snca* mRNA levels as compared to the 20-time winners (U = 41; *p*<0.05). No-fighting losers had decreased *Snca* mRNA levels as compared to the levels in the 20-time losers (U = 30; p<0.01). There were significant differences in the *Snca* mRNA levels between the 20-time winners and 20-time losers (U = 38; *p*<0.05) as well as the no-fighting winners and no-fighting losers (U = 29; *p*<0.01). Differences in mRNA levels between the control and no-fighting winners and between the control and 20-time losers were not definitely significant, but strongly suggestive (p = 0.086 and p = 0.061, respectively). The 20-time winners had increased *Bdnf* mRNA level as compared to the control (U = 22; *p*<0.01). Other pair comparisons failed to reach significance.

Based on Spearman's rank correlation coefficient, there were significant positive correlations in the raphe nuclei between the mRNA levels of *Bdnf* and *Snca* genes in the groups of the 20-time winners and no-fighting winners (R = 0.643, *p*<0.05 and R = 0.734, p<0.01, respectively); in the group of the 20-time losers (R = 0.560, *p*<0.05); and in all groups in combination using pooled data from all the groups (R = 0.397, *p*<0.001). There was one significant positive correlation in the VTA between the mRNA levels of *Bdnf* and *Snca* genes in the control (R = 0.893, *p*<0.01).

## Discussion

It has been shown that long positive fighting experience in daily agonistic interactions is accompanied by activation of brain dopaminergic systems and reduced serotonergic activity in male mice [16]. Chronic social defeat stress is accompanied by activation of the brain serotonergic system and, presumably, decreased dopaminergic activities [17]. Our neurochemical observations in the aggressive mice (winners) are in agreement with other studies in animals and humans [30]–[32].

Two-way ANOVA for the *Snca* mRNA levels in the raphe nuclei and VTA showed significant interaction effects between “social status” and “no-fight period” factors. This means that expression of the *Snca* gene in brain areas changes differently in animals with positive or negative fighting experience and that the changed monoaminergic activity influences gene expression during no-fight period. Increased mRNA levels of the *Snca* gene were found in the 20-time winners' VTA and in the 20-time losers' raphe nuclei in comparison with respective level in opposite social group. On the contrary, decreased *Snca* mRNA level was found in no-fighting winners' VTA and in the no-fighting losers' raphe nuclei in similar comparisons.

Our data suggests that: the changes in *Snca* gene expression are a consequence of the functional state of the brain dopaminergic and serotonergic systems. Enhanced expression of the *Snca* gene due to repeated aggression or defeats is associated with activation of the leading monoaminergic systems: mesolimbic dopaminergic system in the VTA of the 20-time winners and serotonergic system in the raphe nuclei of the 20-time losers. After no-fight period increased *Snca* gene expression in both areas reverts to the control level. On the contrary, when reduced activity of dopaminergic systems in the 20-time losers' VTA or serotonergic system in the 20-time winners' raphe nuclei is suggested, no changes in *Snca* gene expression were found under repeated agonistic interactions. After no-fight period the *Snca* mRNA levels are increased in these areas. Since similar changes in *Snca* gene expression were found in different brain areas, *Snca* may act as a common regulator of monoaminergic activity as shown earlier [3], [4].

Our data provide evidence that the *Snca* gene may be part of a feedback mechanism in regulation of neurotransmitters' metabolism. It has been shown, that the presynaptic protein α-Syn negatively modulates DAT and SERT activity [6], [33]. Increase of the *Snca* mRNA levels may be a response to the increase of *Dat1* mRNA level in the winners' VTA and *Sert* mRNA level in the losers' raphe nuclei shown earlier [12], [13], [18]. After no-fight period mRNA level of *Snca* gene reverts to the control level. Noteworthy, over-expression of the *Snca* gene in the VTA was found in the 20-time winners and in the no-fighting losers in comparison with the control. Some authors suggest [6] that over-expression of the *Snca* gene may block its neuroprotective properties. Our observations are in agreement with those of Mash and co-workers [34] who have demonstrated over-expression of *Snca* gene in the VTA in chronic cocaine abusers, which was shown to activate the brain dopaminergic systems [35]. Increased mRNA level of *Snca* gene was also found after amphetamine injections [36].

Two-way ANOVA for *Bdnf* mRNA levels did not reveal significant interaction effects for “social status” and “no-fight period” in both areas. However, the expression of some genes may increase rapidly and decrease abruptly, while that of other genes changes more gradually [37]. As Miczek and the co-workers report [38], continuous subordination stress leads to significantly decreased levels of BDNF protein in the VTA compared to control levels, whereas intermittent social defeat stress episodes result in increased BDNF protein levels. Thus, the lack of changes in *Bdnf* mRNA levels in the 20-time losers could be explained by transient (dynamic) changes of gene expression shown, for example, for the genes of kappa-opioid receptors [39], [40], mu-opioid receptors [41], [42], and proenkephalin [43] in some brain areas in response to exposure to the experimental settings. If this explanation is correct, we cannot completely exclude the involvement of *Bdnf* in the mechanisms underlying repeated aggression or defeats. This expectation is supported by increased *Bdnf* mRNA level in the 20-time winners' raphe nuclei in comparison with the controls and by the presence of positive functional correlations between the *Bdnf* and *Snca* mRNA levels in the raphe nuclei. These data suggest that BDNF may play an important role in regulation of serotonergic activity. In the VTA, positive correlation between the *Bdnf* and *Snca* mRNA levels was found only in the control mice. However, the intrinsic molecular mechanisms responsible for the functional association have yet to be revealed. The reason for this correlative relationship might be the common molecular mechanisms of transcriptional regulation of these genes.

Thus, chronic manifestation of aggression, which leads to activation of dopaminergic metabolism in the brain areas, enhances in the VTA the expression of the *Th, Dat1 and Snca* genes [13], whose proteins are responsible for the DA functioning. Mesolimbic dopaminergic projections from the VTA play an important role in the mediation of rewarding processes. It is therefore possible that the observed changes of the *Snca* genes expression display the dopaminergic mechanisms from experiencing positive emotions over social victories in the winners. Because social defeats lead to the activation of the serotonergic system [17], [44], the changes in the *Snca* mRNA levels in the losers' raphe nuclei lend support to the involvement of a-Syn in the consequence of chronic negative emotions.

It has been shown earlier that long positive fighting history leads to development of behavioral psychopathology, which includes the demonstration of abnormal aggression, malignancy, strong hostility, pronounced anxiety, disturbances in social recognition, hyperactivity, stereotypic and hyperkinetic reactions [16]. Male mice with long defeat history developed a psychoemotional disorder similar to anxious depression in accordance with symptomatics, etiology factors, brain neurochemical changes and sensitivity to antidepressants and anxiolytics similar to those in depressive persons [17], [44]. It may be concluded that *Snca* gene may be involved in pathogenesis of these disorders. In this context our assumption is in agreement with earlier reports [8], [45], [46] which have demonstrated that the ability of α-Syn to modulate SERT and DAT functions may be of pathological significance, particularly with regard to psychiatric disorders such as depression, suicide, and impulsive violence.

It must be noted that the activities of all neurotransmitter systems may be dynamically changed as for metabolism, receptors and enzyme activities in male mice in response to chronic activation or inhibition of neurotransmitter' systems depending on social status and/or duration of repeated agonistic interactions [16], [17]. Our behavioral approach makes it possible to track changes in gene functioning during development of behavioral pathologies [47], and to study the transcriptional state of a set of genes, which may be involved in the brain pathogenesis.
